# The evolution of standards and data management practices in systems biology

**DOI:** 10.15252/msb.20156053

**Published:** 2015-12-28

**Authors:** Natalie J Stanford, Katherine Wolstencroft, Martin Golebiewski, Renate Kania, Nick Juty, Christopher Tomlinson, Stuart Owen, Sarah Butcher, Henning Hermjakob, Nicolas Le Novère, Wolfgang Mueller, Jacky Snoep, Carole Goble

**Affiliations:** ^1^ Manchester Institute of BiotechnologyThe University of ManchesterManchesterUK; ^2^ School of Computer ScienceUniversity of ManchesterManchesterUK; ^3^ Leiden Institute of Advanced Computer Science, Leiden Institute of Advanced Computer ScienceLeiden UniversityLeidenThe Netherlands; ^4^ Heidelberg Institute for Theoretical StudiesHeidelbergGermany; ^5^ European Molecular Biology LaboratoryEuropean Bioinformatics Institute (EMBL‐EBI)CambridgeUK; ^6^ Department of Surgery and CancerImperial College LondonLondonUK; ^7^ Babraham InstituteCambridgeUK; ^8^ Department of BiochemistryUniversity of StellenboschMatielandSouth Africa; ^9^ School of Chemical Engineering & Analytical ScienceThe University of ManchesterManchesterUK

**Keywords:** Methods & Resources

## Abstract

A recent community survey conducted by Infrastructure for Systems Biology Europe (ISBE) informs requirements for developing an efficient infrastructure for systems biology standards, data and model management.

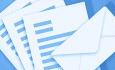

## Introduction

Systems biology involves the integration of multiple heterogeneous data sets, in order to model and predict biological processes. The domain's interdisciplinary nature requires data, models and other research assets to be formatted and described in standard ways to enable exchange and reuse.

Infrastructure for Systems Biology Europe (ISBE) is a project to establish essential, centralized services for systems biology researchers throughout the systems biology lifecycle. A key component of ISBE is to support the management, integration and exchange of data, models, results and protocols. To inform further ISBE development, we surveyed the community to evaluate the uptake of available standards, and current practices of researchers in data and model management.

The survey addressed four key areas as follows:

1. Standards usage;
2. Data and model storage before publication;
3. Sharing in public repositories after publication;
4. Reusability of data, models and results.

The survey was sent to major mailing lists targeting the systems biology and computational biology communities and advertised at relevant consortia meetings. It elicited 153 responses, from 17 countries across 6 continents, with a cross section of the systems biology community represented (Appendix Fig S1). Lessons from the survey are being implemented as part of an ISBE supporting project, FAIRDOM (www.fair-dom.org).

**Figure 1:**
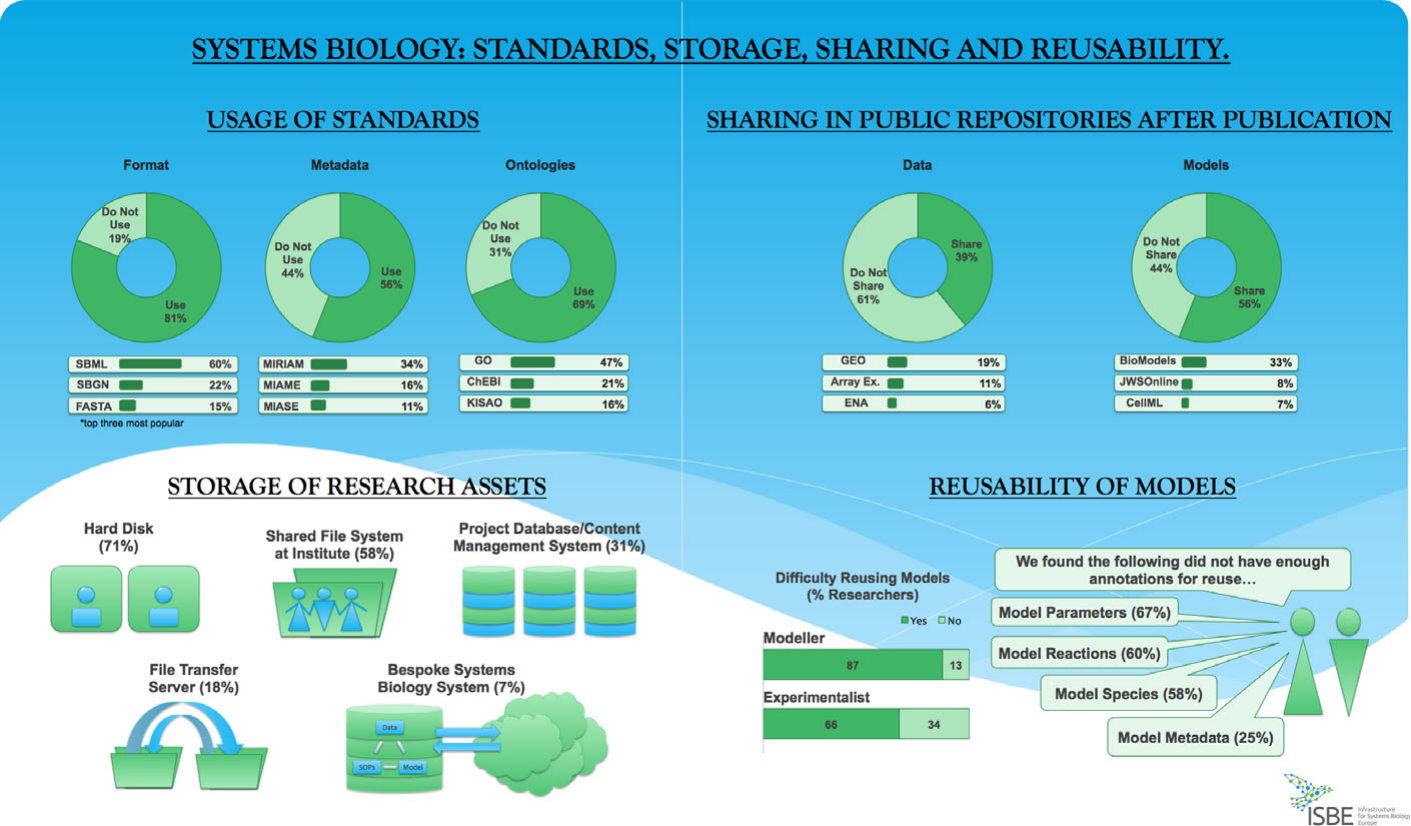
Survey summary.

To understand how uptake of standards has developed, we compared our findings to a previous study by Klipp *et al* in 2007. Fig 1 shows a summary of the survey results (detailed results in Dataset EV1). A number of acronyms are used within the text, details of which can be found in Table 1.

**Table 1:** Glossary of acronyms

Acronym	Description	Link
Array Ex.	Array Express—archive of functional genomics data	https://www.ebi.ac.uk/arrayexpress/
BioModels	Database for storing curated and non‐curated systems biology computational models	https://www.ebi.ac.uk/biomodels/
CellML	Standard for formatting models, as well as a model repository	https://www.cellml.org/
ChEBI	Chemical Entities of Biological Interest—a dictionary of molecular entities	https://www.ebi.ac.uk/chebi/init.do
COMBINE	Computational Modelling in Biology Network	http://co.mbine.org
ENA	European Nucleotide Archive—a comprehensive record of nucleotide sequences	http://www.ebi.ac.uk/ena
FAIRDOM	Findable Accessible Interoperable Reusable Data standard Operating Procedures and Models	http://fair-dom.org
FASTA	Text‐based format for representing nucleotide sequences	https://en.wikipedia.org/wiki/FASTA_format
GEO	Gene Expression Omnibus—repository for functional genomics data	http://www.ncbi.nlm.nih.gov/geo/
GO	Gene Ontology—a controlled vocabulary of gene and gene product attributes	http://geneontology.org/
ISBE	Infrastructure for Systems Biology Europe	http://project.isbe.eu
ISO	International Standards Organization	http://www.iso.org
JWS Online	Tool for online simulation of systems biology models	http://jjj.mib.ac.uk/
KISAO	Kinetic Simulation Algorithm Ontology, for identifying algorithms and associated set‐up of simulations	http://co.mbine.org/standards/kisao
MIAME	Minimum Information about a Microarray Experiment	http://fged.org/projects/miame/
MIASE	Minimum Information about a Simulation Experiment	http://co.mbine.org/standards/miase
MIRIAM	Minimum Information Required in the Annotation of Models	http://co.mbine.org/standards/miriam
SBGN	Systems Biology Graphical Notation	http://www.sbgn.org/
SBML	Systems Biology Mark‐up Language	http://sbml.org/
SEEK	Bespoke systems biology data management platform, which works as an aggregated content commons, and a database	http://fair-dom.org/SEEK

## Standards usage

Formatting and describing data and models using community standards enables them to be understood, compared, exchanged and reused by both collaborators and the wider community. As such, uptake of standards is vital for high‐quality, reproducible research. This is especially true for systems biology which naturally requires frequent exchange of data and models. In systems biology, standards are primarily developed by community standardization initiatives such as COMBINE (Hucka *et al*, 2015), and ISO.

In this study, we consider three major types of standards as follows:

1. Standard formats for representing data and models;
2. Standard metadata checklists for describing particular types of data and models;
3. Controlled vocabularies and ontologies to provide a common notation and annotation vocabulary.

In 2007, Klipp *et al* identified formats, in particular those for encoding models, as the most widely used standards. This is still the case now, with SBML (60%) and SBGN (22%) (Hucka *et al*, 2015) dominating. These standard formats allow easy exchange between software tools and databases, improving (re)usability. The availability and uptake of formats has grown rapidly since 2007. Standards for formatting and visualizing models and for some common experimental data are now available.

Metadata standards—standards for data describing the data—were highlighted as requiring significant development in 2007. There are now over 40 *minimum information* checklists that consistently structure the least amount of information required to interpret a data set. These include common data and model types in systems biology (see Appendix). MIRIAM (Le Novère *et al*, 2005), MIAME (Brazma *et al*, 2001) and MIASE (Waltemath *et al*, 2011) are the most used by respondents. Ontologies are often used as annotation vocabularies within metadata descriptions. Ontologies for annotating gene functions (GO—47% Ashburner *et al*, 2000), small molecules (ChEBI—21% Hastings *et al*, 2013) and model simulations (KISAO—16% Courtot *et al*, 2011) are the most popular in the community, with growing acceptance since 2007.

Whilst the availability of standards and their growing uptake is encouraging, there is still a dearth of standards for many data types. A priority must be to increase standard availability for common data types not covered. One of the major bottlenecks for uptake is most likely the lack of tools that implement support for standards. If standards compliant results were supported by information management software, it would become part of the research process and thereby reduce the time, knowledge and skills required to achieve compliance, facilitating quicker and more widespread adoption.

## Storage of research assets

Systems biology researchers need to exchange experimental data, computer code and models between collaborators within their institute and with distributed, external partners. Despite this exchange being a key activity, the majority of researchers still only store their work on their local hard disc (71%), or shared file systems within their institute (58%). This can make versioning or snapshotting research assets difficult and raises barriers for sharing with collaborators, or, for example, when key personnel leave a team. Content management systems and bespoke systems biology platforms are more amenable to organizing, versioning and sharing, but are only used by 31% and 7% of researchers, respectively. Bespoke platforms require more investment in upload and updating, but provide users with more security for data backup, and offer versioning and easier sharing options.

## Sharing in public repositories

Using public repositories is more common to share models (56%) than data (39%). BioModels (Chelliah *et al*, 2015) is the most popular models database (33%)—it is also one of the most popular for finding models after publication (22%). Data are often published in dedicated repositories, grouped by data type (e.g. metabolomics data in a metabolomics database), rather than by function (e.g. all data on human liver). This can make identifying complementary datasets for integration into models difficult, even if the data are well annotated. A major disadvantage for systems biology results is that data sets that were generated from the same samples to address specific biological processes can be separated and submitted to several independent repositories, which results in a loss of experimental context. Some researchers use content aggregator commons, such as SEEK (7%) (Wolstencroft *et al*, 2015), which support functional linking for data and model integration, helping retain experimental context.

Sharing data and models solely through supplementary material in journal articles is still common practice. This represents a publication‐centric view of the data, which means finding related data might be more difficult than it would be when data are submitted to public repositories.

## Reusability of models

Being able to reuse data and models in different studies allows a maximized return on research investments. The majority of respondents found it difficult to reuse models and associated data. Model parameters and the traceability of their origins were particularly notable as areas that needed improvement (67% finding issues). These could be improved with better annotation of the original data and better semantic linking of the models to the experimental data that was used to construct them.

## Conclusions and outlook

It is clear from the research that we need:

1. Software tools that support standards, thereby facilitating their adoption;
2. Shared/cloud‐based platforms to disseminate assets across the community;
3. Annotate and curate assets to enable their meaningful integration;
4. Intimately and persistently, link structured and annotated data and models.

To address the issues above, we suggest that centralized coordinated infrastructures like ISBE, in collaboration with standardization initiatives such as COMBINE, take lead in improving availability, adoption and long‐term sustainability of standards. This can be achieved through the training of researchers as well as tool development to support their work flows. The community should also look towards encouraging data and model sharing through incentives such as credit mechanisms and appropriate mandates on practices from journals.

## Supporting information



Appendix

Dataset EV1
