# Supplementary material for: The evolution of standards and data management practices in systems biology
Source: Mol Syst Biol. 2015 Dec 28;11(12):851. doi: 10.15252/msb.20156053 (PMC4704484; doi:10.15252/msb.20156053)
Supplement: Supplementary file 1 — Appendix [file MSB-11-851-s001.pdf]

ISBE STANDARDS SURVEY RESULTS 2015

[1,2*]Natalie J Stanford, [3]Katherine Wolstencroft, [4]Martin Golebiewski, [4]Renate Kania, [5]Nick Juty, [6]Christopher Tomlinson, [2]Stuart Owen, [6]Sarah Butcher, [5]Henning Hermjakob, [7]Nicolas Le Novère, [5]Wolfgang Mueller, [8,9]Jacky Snoep, [2]Carole Goble.

ISBE Infrastructure for Systems Biology Europe

1 Manchester Institute of Biotechnology, The University of Manchester, 131 Princess Street, Manchester, M1 7DN, United Kingdom; 2 School of Computer Science, University of Manchester, Kilburn Building, Oxford Road, Manchester M13 9PL, United Kingdom; 3 Leiden Institute of Advanced Computer Science Leiden Institute of Advanced Computer Science, Leiden University, 111 Snellius, Niels Bohrweg 1, 2333 CA Leiden, Netherlands; 4 Heidelberg Institute for Theoretical Studies, Schloss-Wolfsbrunnenweg 35 69118 Heidelberg, Germany; 5 European Bioinformatics Institute (EMBL-EBI), European Molecular Biology Laboratory, Wellcome Trust Genome Campus, Hinxton, Cambridge. CB10 1SD; 6 Department of Surgery and Cancer, Imperial College London, South Kensington, London, SW72AZ; 7 Babraham Institute, Babraham Research Campus, Cambridge CB22 3AT, united-Kingdom; 8 Department of Biochemistry, University of Stellenbosch, Private Bag X1, 7602 Matieland, South Africa; 9 School of Chemical Engineering & Analytical Science, The University of Manchester, Oxford Road, Manchester, M13 9PL, United Kingdom.

Contents

List of Figures:	Page
Figure S1: Shows the geographical, and discipline distribution of respondents.	2
Figure S2: Here we show the percentage of respondents who do and do not use standard formats, standard metadata descriptions, and ontologies, as well as the most popular standards used.	3
Figure S3: Respondents detail difficulties they have faced when implementing standards in their research.	4
Figure S4: Respondents more frequently store unpublished research assets on local hard disks, or shared file systems at their institute.	5
Figure S5: Models are shared in public repositories after publication more often than data are. The most popular repositories for sharing data and models are detailed.	6
Figure S6: The most popular repositories for finding data.	7
Figure S7: Most respondents had difficulty reusing models, describing provenance of parameters in models as being a major contributor to the lack of reusability.	8

List of Tables:	Page
Table S1: Public repositories mentioned	10
Table S2: Data and model standards mentioned	11
Table S3: Metadata standards mentioned	12
Table S4: Ontologies mentioned	13

RESPONDENT DEMOGRAPHICS

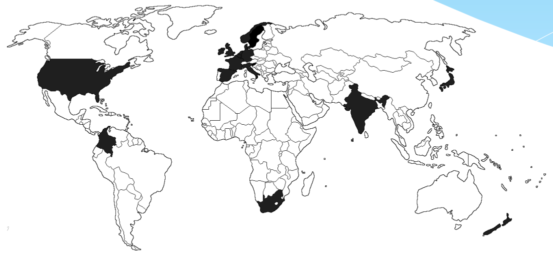

153 Respondents
17 Countries
6 Continents

Respondents came from a range of backgrounds, and as expected identify as experts in more than one role

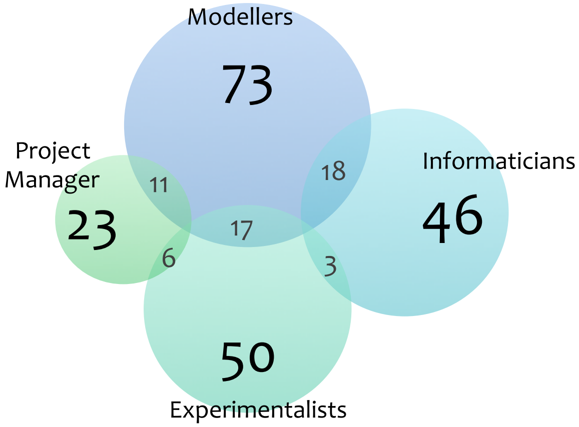

Figure S1: Shows the geographical, and discipline distribution of respondents.

USAGE OF STANDARDS

Format

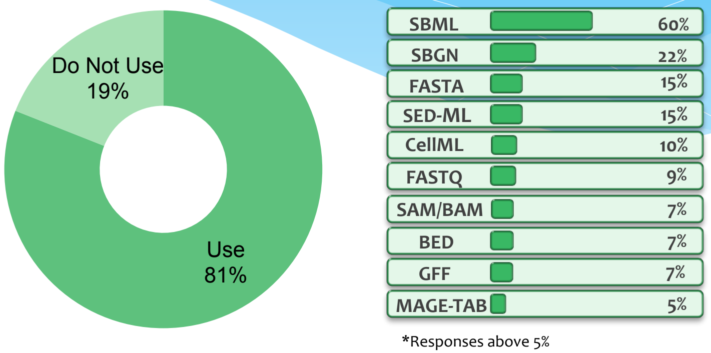

*Responses above 5%

Metadata

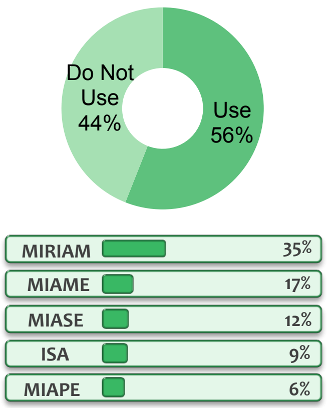

Ontologies

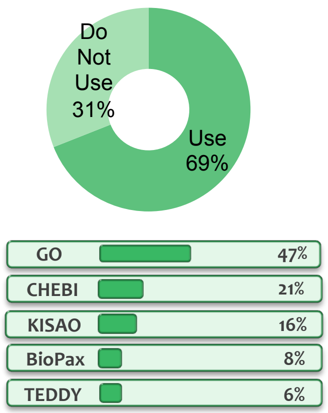

Figure S2: Formats show the most uptake among respondents, followed by ontologies, and metadata. Standards for models are among the most popular used.

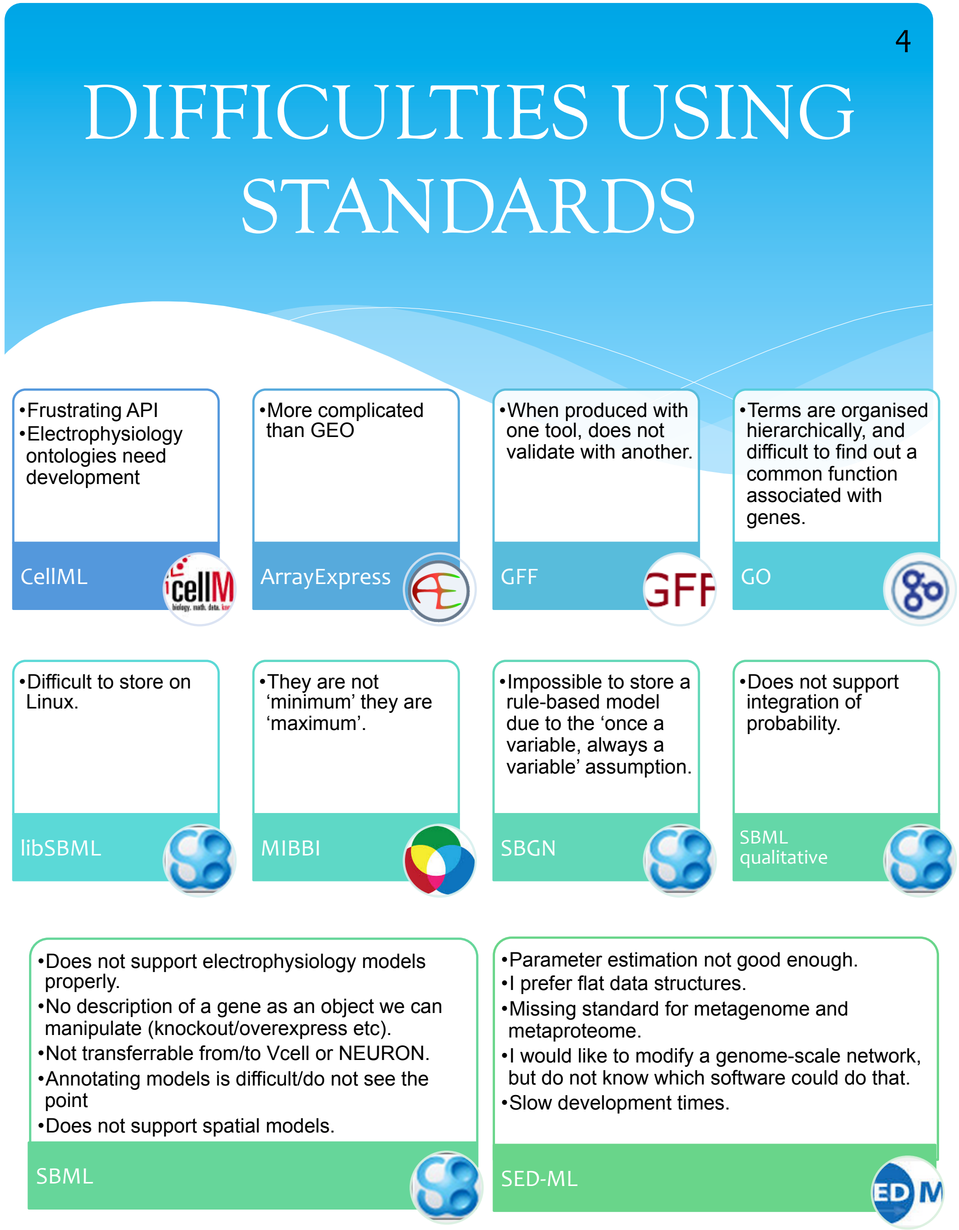

Figure S3: Respondents detail difficulties they have faced when implementing standards in their research.

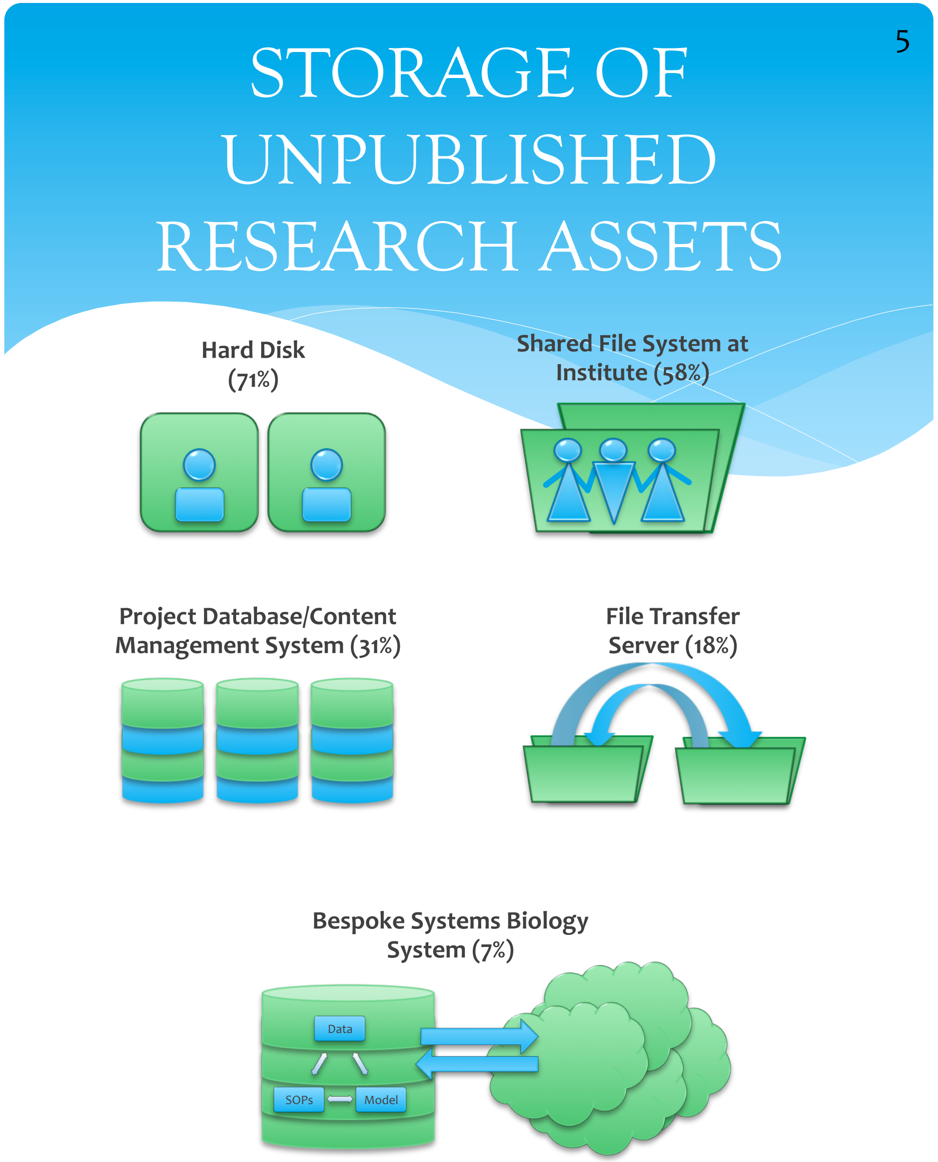

Figure S4: Respondents more frequently store unpublished research assets on local hard disks, or shared file systems at their institute.

SHARING IN PUBLIC REPOSITORIES AFTER PUBLICATION

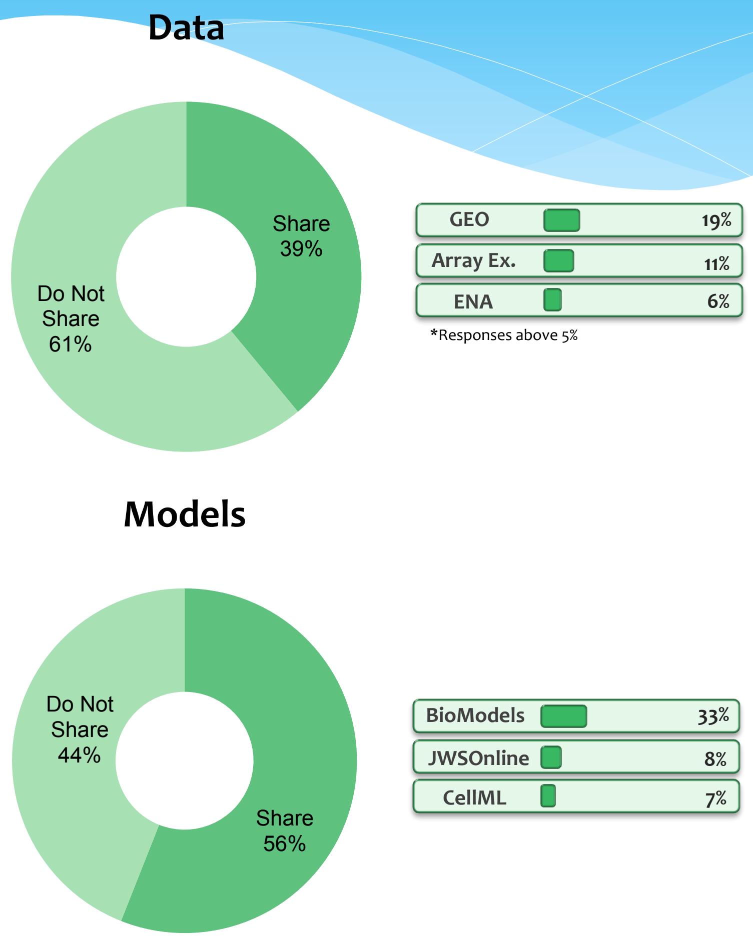

Figure S5: Models are shared in public repositories after publication more often than data are. The most popular repositories for sharing data and models are detailed.

REPOSITORIES USED FOR FINDING DATA

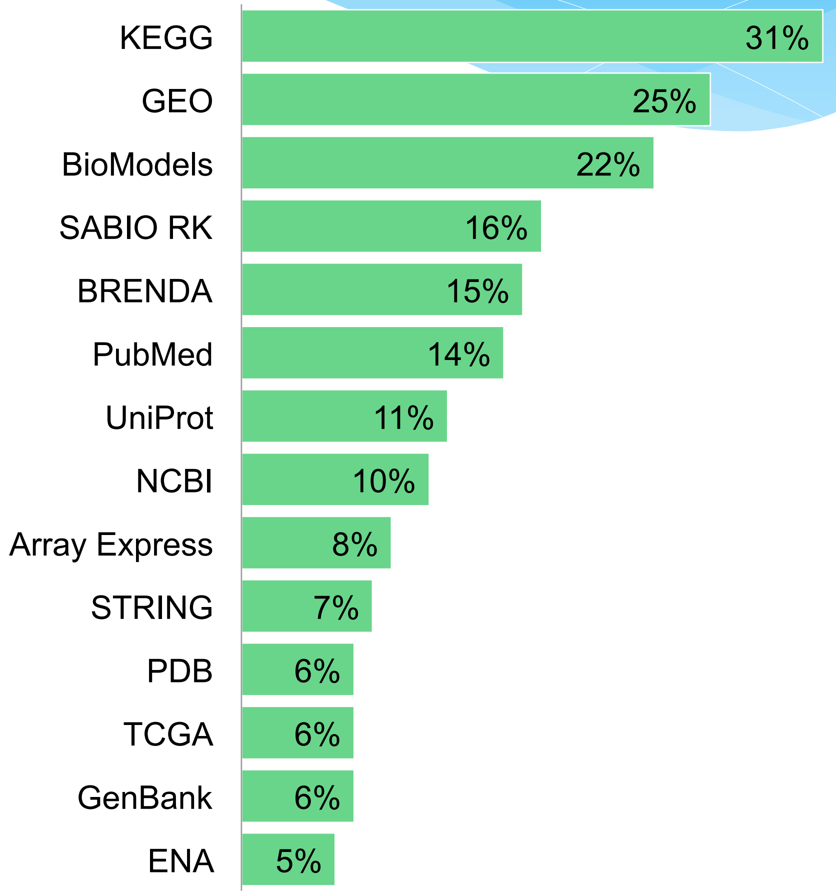

Figure S6: The most popular repositories for finding data.

REUSABILITY OF MODELS

Difficulty Re-using Models:

YES NO

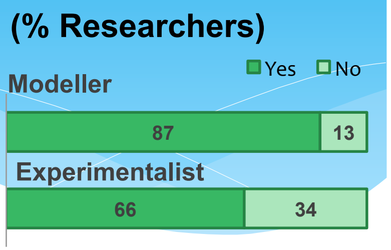

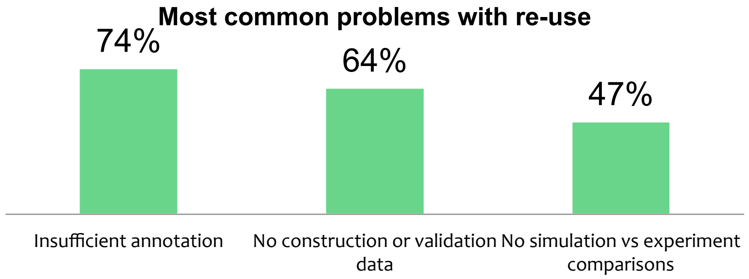

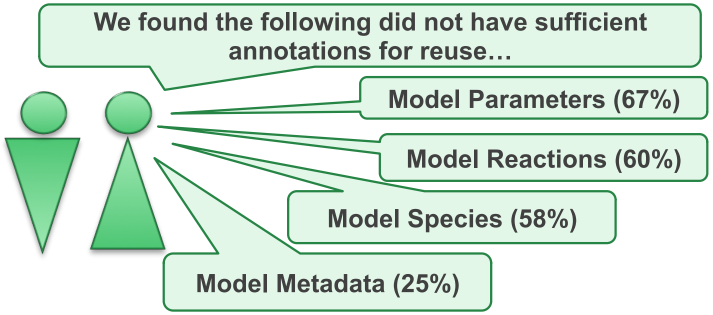

Figure S7: Most respondents had difficulty reusing models, describing provenance of parameters in models as being a major contributor to the lack of reusability.

APPENDIX

TABLE S1: PUBLIC REPOSITORIES MENTIONED

Resource	Description	URL
**Array Express**	Array Express – archive of functional genomics data.	https://www.ebi.ac.uk/arrayexpress/
**BiGG**	A biochemical, genetic and genomic knowledge base for generating large-scale metabolic reconstructions.	http://bigg.ucsd.edu/
**BioCyc**	Collection of databases for different cellular functions	http://biocyc.org/
**BioModels**	For storing SBML models.	http://www.ebi.ac.uk/biomodels-main/
**BioUML**	Platform for analysing 'omics data using computational biology tools.	http://wiki.biouml.org/index.php/BioUML_wiki
**BRENDA**	The Comprehensive Enzyme Information Systems.	http://www.brenda-enzymes.org/
**CellML Model repository**	For storing CellML models.	https://models.cellml.org/cellml
**ENA**	European Nucleotide Archive – a comprehensive record of the worlds nucleotide sequences.	http://www.ebi.ac.uk/ena
**GenBank**	National Institute of Health genetic sequence database.	http://www.ncbi.nlm.nih.gov/genbank/
**GEO**	Gene Expression Omnibus – repository for functional genomics data.	http://www.ncbi.nlm.nih.gov/geo/
**insilicoDB**	Subscription based modelling solution.	https://insilicodb.com/
**JWS Online**	For storing SBML models, as well as an online simulation environment.	http://jjj.biochem.sun.ac.za
**KEGG**	KEGG is a database resource for understanding high-level functions and utilities of the biological system.	http://www.genome.jp/kegg/
**MetaCrop**	Summary of information relating to metabolic pathways in crop plants.	http://metacrop.ipk-gatersleben.de/
**Model DB**	Yale resource for model sharing, no specified formats, and shows flexibility for storing parameter sets.	http://senselab.med.yale.edu/modeldb/mdb_model_sharing.asp
**Open Source Brain**	For collaborative development of brain models.	http://www.opensourcebrain.org/
**wwPDB**	World Wide Protein Data Bank – holds information on 3D structures of proteins.	http://www.wwpdb.org/
**PubMed**	For biomedical literature.	http://www.ncbi.nlm.nih.gov/pubmed
**Sabio-RK**	A curated database containing information about biochemical reactions – including kinetic rate equations, parameters, experimental conditions.	http://sabio.villa-bosch.de/
**SEEK**	Commons resource for storing research assets in ISA format.	http://www.seek4science.org/
**STRING**	For known and predicted protein-protein interactions.	http://string-db.org/
**TCGA**	The Cancer Genome Atlas.	http://cancergenome.nih.gov/
**UniProt**	For protein sequence and functional information.	http://www.uniprot.org/
**Virtual Cell**	Internet based modelling software for mathematical models in general.	http://www.nrcam.uchc.edu/

TABLE S2: DATA AND MODEL STANDARDS MENTIONED

Resource	Description	URL
**BED**	Text file that defines a feature track of sequence data.	http://genome.ucsc.edu/FAQ/FAQformat#format1
**CellML**	For structuring computer based mathematical models	https://www.cellml.org/
**FASTA**	A text based format for representing nucleotide sequences or peptide sequences.	http://www.ncbi.nlm.nih.gov/BLAST/blastcgihelp.shtml
**FASTQ**	A text based format for storing both biological sequences and corresponding quality scores.	http://maq.sourceforge.net/fastq.shtml
**GEL-ML**	For protein separation experiments.	http://www.psidev.info/gelml/1.0
**GFF**	Exchange format for feature description of sequence data.	https://www.sanger.ac.uk/resources/software/gff/
**KGML**	KEGG Markup Language for exchange pathway maps.	http://www.kegg.jp/kegg/xml/docs/
**MAGE-ML**	MicroArray Gene Expression Markup Language – for structuring gene expression data.	https://www.biosharing.org/bsg-000573
**MAGE-TAB**	Spreadsheet format for structuring gene expression data.	http://fged.org/projects/mage-tab/
**MathML**	Low level specification for mathematical and scientific content on the web.	https://www.w3.org/Math/
**mmCIF**	For representing macromolecular structural data.	http://www.ebi.ac.uk/pdbe/docs/documentation/mmcif.html
**MzML**	For structuring raw spectrometry output.	http://www.psidev.info/mzml_1_0_0%20
**NeuroML**	For structuring computational neuroscience models.	https://www.neuroml.org/
**OME-XML**	For the exchange of microscopy imaging data.	https://www.openmicroscopy.org/site/support/ome-model/ome-xml/
**PharmML**	For encoding models for pharmacometric models.	http://www.pharmml.org/
**SAM/BAM**	Sequence Alignment Map – for storing large nucleotide sequence maps (BED is the binary form of SAM).	http://samtools.sourceforge.net/
**SBGN**	Systems Biology Graphical Notation – for visualizing systems biology models.	http://www.sbgn.org/Main_Page
**SBML**	Systems Biology Markup Language – for structuring of systems biology models.	http://sbml.org/Main_Page
**SBRML**	Systems Biology Results Mark-up Language – for structuring simulation results.	http://www.comp-sys-bio.org/SBRML.html
**SED-ML**	Simulation Experiment Description Markup Language – for structuring simulation descriptions for reproduction.	http://sed-ml.org/

TABLE S3: METADATA STANDARDS MENTIONED

Resource	Description	URL
**CIMR**	Core Information for Metabolomics Reporting.	http://mibbi.sourceforge.net/projects/CIMR.shtml
**ISA**	Investigation Study Assay.	http://isatab.sourceforge.net/format.html
**MIACA**	Minimum Information about a Cellular Assay.	http://miaca.sourceforge.net/
**MIAME**	Annotation of sequence data from microarray experiments.	http://www.ncbi.nlm.nih.gov/pubmed/11726920
**MIAPE**	Minimum Information about Proteomics Experiment.	http://www.psidev.info/node/91
**MIARE**	Minimum Information about an RNAi Experiment.	http://miare.sourceforge.net/HomePage
**MIASE**	Minimumum information about a simulation experiment, allowing the replication of computational simulations.	http://co.mbine.org/standards/miase
**MIFlowCyt**	Minimum Information about a Flow Cytometry Experiment	http://flowcyt.sourceforge.net/miflowcyt/
**MIGen**	Minimum Information about a Genotyping Experiment	http://migen.sourceforge.net/
**MIMIx**	Minimum Information required for reporting a Molecular Interaction eXperiment	http://www.psidev.info/node/278
**MINSEQE**	Minimum Information about high-throughput nucleotide SEQuencing Experiment.	http://fged.org/projects/minseqe/
**MIQE**	Minimum Information for Publication of Quantitative Digital PCR Experiments	http://www.miqe.info/
**MIRIAM**	Minimum guidelines for computational models annotation and curation.	http://co.mbine.org/standards/miriam
**STRENDA**	STandards for REporting ENzyme DAta	http://www.beilstein-institut.de/en/projects/strenda/aims

TABLE S4: ONTOLOGIES MENTIONED

Resource	Description	URL
**BioPax**	Biological Pathway Exchange: for describing pathway data.	http://www.biopax.org/
**CHEBI**	Chemical Entities of Biological Interest: used for describing 'small' chemicals.	http://www.ebi.ac.uk/chebi/
**EC numbers**	Enzyme Comission Number – a numerical classification systems for enzymes.	https://en.wikipedia.org/wiki/Enzyme_Commission_number
**ECO**	The Evidence Ontology: for describing scientific evidence in the realm of biology.	http://www.evidenceontology.org/Welcome.html
**EMO**	Enzyme Mechanism Ontology: for describing enzyme mechanisms.	http://bio-ontologies.knowledgeblog.org/176
**GO**	Gene Ontology: used for describing gene products.	http://geneontology.org/
**Identifiers.org**	Providing persistent URIs for biological data.	http://identifiers.org/
**JERM**	Just Enough Results Model: for exchange, interpretation, and comparison between different types of data and results.	http://www.sysmo-db.org/jerm
**KEGG**	Kyoto Encyclopedia of Genes and Genomes. Used for molecular level information.	http://www.genome.jp/kegg/
**KISAO**	Kinetic Simulation Algorithm Ontology: for describing kinetic simulations.	http://biomodels.net/kisao/
**MeSH**	Medical Subject Headings: used for indexing medical papers.	http://www.nlm.nih.gov/mesh/meshhome.html
**MGED**	For annotation of microarray experiments.	http://co.mbine.org/standards/teddy/ontology
**MICEE**	Minimum Information about a Cardiac Electrophysiology Experiment: for annotation of documents describing cardiac electrophysiological experiments.	https://www.micee.org/
**NeuroLex**	Lexicon of neuroscience terms.	http://neurolex.org/wiki/Main_Page
**OPB**	Ontology of Physics for Biology: for classical physics as applied to biological systems.	http://sbp.bhi.washington.edu/projects/the-ontology-of-physics-for-biology-opb
**PSI_MOD**	Proteomic Standards Initiative: for describing protein modifications.	http://www.psidev.info/MOD
**PSI_MS**	Proteomics Standards Initiative: for describing protein-protein interactions.	http://www.psidev.info/
**RNAO**	RNA Ontology: for RNA function in biological systems.	http://roc.bgsu.edu/
**SBO**	Systems Biology Ontology – for terms used particularly in computational modelling.	http://www.ebi.ac.uk/sbo/main/
**SO**	Sequence Ontology: for the definition of sequence features.	http://www.sequenceontology.org/
**SWO**	Software Ontology	http://theswo.sourceforge.net/
**TEDDY**	For describing observable dynamical behaviours, and control.	http://co.mbine.org/standards/teddy/ontology
