# Supplementary material for: The evolution of standards and data management practices in systems biology
Source: Mol Syst Biol. 2015 Dec 28;11(12):851. doi: 10.15252/msb.20156053 (PMC4704484; doi:10.15252/msb.20156053)
Supplement: Supplementary file 2 — Dataset EV1 [file MSB-11-851-s002.pdf]

ISBE Systems Biology Standards Survey

Q3 What is your scientific background?

Answered: 136   Skipped: 16

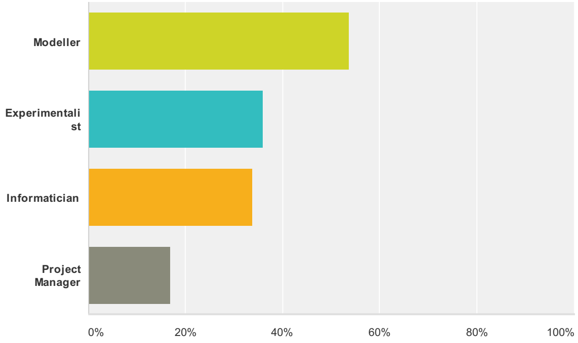

Answer Choices	Responses	
Modeller	**53.68%**	73
Experimentalist	**36.03%**	49
Informatician	**33.82%**	46
Project Manager	**16.91%**	23
**Total Respondents: 136**		

#	Other (please specify)	Date
1	PhD student	10/7/2013 9:20 AM
2	Group leader	10/4/2013 10:59 AM
3	Computer Science / Computational Physiology	9/30/2013 10:00 AM
4	biosystems engineering	9/26/2013 9:25 AM
5	Technical writer	9/25/2013 11:16 PM
6	Biochemist	9/25/2013 5:56 PM
7	data management and integration specialist	9/25/2013 2:01 PM
8	Software & standards development	9/25/2013 1:20 PM
9	Engineer	9/20/2013 10:01 AM
10	Computer science	9/19/2013 11:32 AM
11	Computer Scientist	9/19/2013 10:30 AM
12	Grants Preparation	9/4/2013 11:43 AM
13	phd student biologist doing now more algorithms development	9/4/2013 11:37 AM
14	bioinformatics	9/4/2013 11:35 AM
15	Curator	8/2/2013 4:49 PM

ISBE Systems Biology Standards Survey

Q4 Which public repositories do you submit your data to when your work is published? (Please select all that apply).

Answered: 124   Skipped: 28

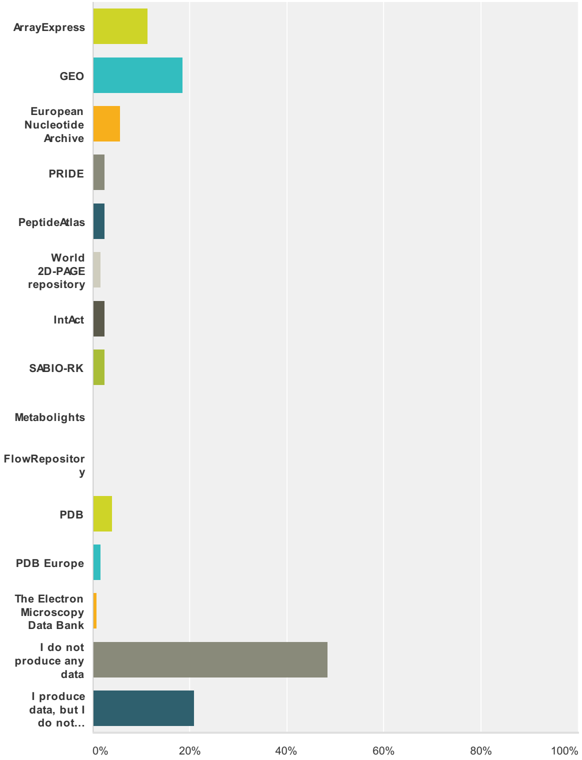

Answer Choices	Responses	
Array Express	11.29%	14
GEO	18.55%	23
European Nucleotide Archive	5.65%	7
PRIDE	2.42%	3
PeptideAtlas	2.42%	3
World 2D-PAGE repository	1.61%	2
IntAct	2.42%	3
SABIO-RK	2.42%	3
Metabolights	0%	0
FlowRepository	0%	0
PDB	4.03%	5
PDB Europe	1.61%	2
The Electron Microscopy Data Bank	0.81%	1
I do not produce any data	48.39%	60
I produce data, but I do not submit it to public repositories	20.97%	26
**Total Respondents: 124**		

#	Other (please specify)	Date
1	SRA	10/2/2013 12:37 PM
2	SRA	10/1/2013 11:47 AM
3	SEEK	9/26/2013 7:45 AM
4	SysMO-SEEK	9/25/2013 3:44 PM
5	TriTrypDB	9/25/2013 2:54 PM
6	Own project website	9/25/2013 2:47 PM
7	VLN	9/25/2013 1:30 PM
8	I faciliate redistribution/access to data	9/19/2013 4:19 PM
9	SEEK	9/18/2013 5:29 PM
10	REACTOME	9/4/2013 11:49 AM
11	cobra	9/4/2013 11:39 AM
12	NCBI SRA	9/4/2013 11:20 AM

Q5 Which public repositories do you submit your models to when your work is published? (Please select all that apply).

Answered: 120 Skipped: 32

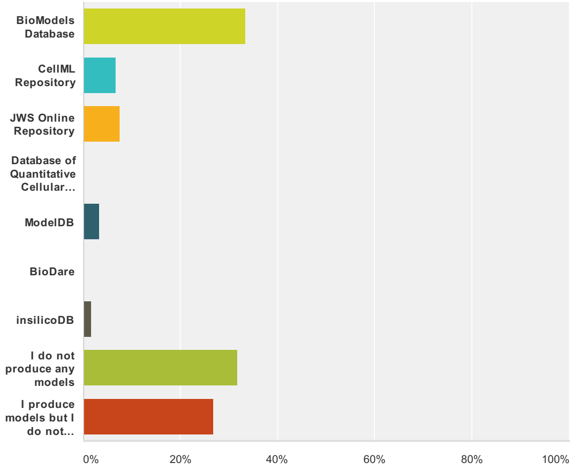

Answer Choices	Responses	
BioModels Database	33.33%	40
CellML Repository	6.67%	8
JWS Online Repository	7.50%	9
Database of Quantitative Cellular Signalling	0%	0
ModelDB	3.33%	4
BioDare	0%	0
insilicoDB	1.67%	2
I do not produce any models	31.67%	38
I produce models but I do not submit them to public repositories	26.67%	32
Total Respondents: 120		

#	Other (please specify)	Date
1	BiGG Database	9/26/2013 11:44 AM
2	I put the models in the supplement of my papers	9/26/2013 11:18 AM
3	SEEK	9/26/2013 7:45 AM
4	transsys.net	9/25/2013 7:00 PM
5	BioCyc	9/25/2013 5:12 PM
6	SysMO SEEK	9/25/2013 3:49 PM
7	Virtual Cell Database	9/25/2013 2:51 PM
8	MetaCrop (more for metabolic pathways than for models)	9/25/2013 1:24 PM
9	Open Source Brain	9/25/2013 1:22 PM
10	BioUML repository	9/19/2013 5:48 PM
11	Model Graphs (internal Graph Database at University of Rostock, development stage)	9/18/2013 5:29 PM
12	SysMO SEEK	9/5/2013 1:27 AM
13	VCell	9/4/2013 4:32 PM
14	I haven't submitted any till now	9/4/2013 11:36 AM
15	not yet	9/4/2013 11:29 AM

ISBE Systems Biology Standards Survey

Q6 What formats do you use for structuring and annotating your data and models? (Please name all that apply).

Answered: 124 Skipped: 28

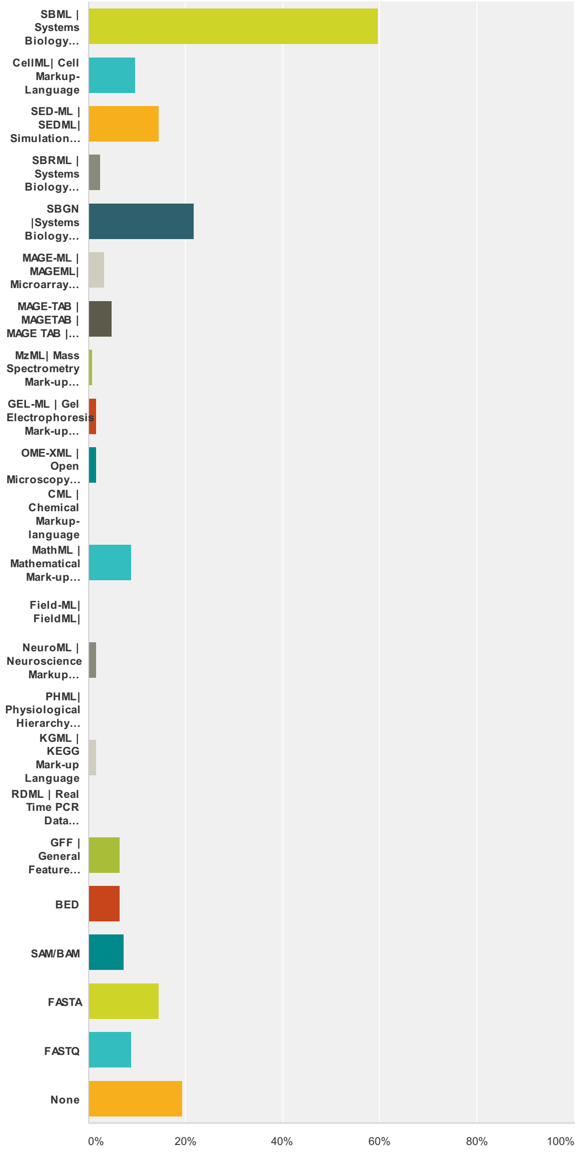

Answer Choices	Responses	
SBML | Systems Biology Mark-up Language	59.68%	74
CellML| Cell Markup-Language	9.68%	12
SED-ML | SEDML| Simulation Experiments Mark-up Language	14.52%	18
SBRML | Systems Biology Results Mark-up Language	2.42%	3
SBGN |Systems Biology Graphical Notation	21.77%	27
MAGE-ML | MAGEML| Microarray Gene Expression Mark-up Language	3.23%	4
MAGE-TAB | MAGETAB | MAGE TAB | Microarray Gene Expression Tabular Format	4.84%	6
MzML| Mass Spectrometry Mark-up Language	0.81%	1
GEL-ML | Gel Electrophoresis Mark-up Language	1.61%	2
OME-XML | Open Microscopy Environment eXtensible Mark-up Language	1.61%	2
CML | Chemical Markup-language	0%	0
MathML | Mathematical Mark-up Language	8.87%	11
Field-ML| FieldML|	0%	0
NeuroML | Neuroscience Markup Language	1.61%	2
PHML| Physiological Hierarchy Mark-up Language	0%	0
KGML | KEGG Mark-up Language	1.61%	2
RDML | Real Time PCR Data Markup-Language	0%	0

GFF | General Feature Format	**6.45%**	8
BED	**6.45%**	8
SAM/BAM	**7.26%**	9
FASTA	**14.52%**	18
FASTQ	**8.87%**	11
None	**19.35%**	24
**Total Respondents: 124**		

#	Other (please specify)	Date
1	Also some in-house formats	9/30/2013 10:03 AM
2	Plain text files	9/26/2013 11:18 AM
3	transsys	9/25/2013 7:00 PM
4	VCML	9/25/2013 2:51 PM
5	PDB, CIF, mmCIF	9/25/2013 2:50 PM
6	MATLAB	9/25/2013 1:22 PM
7	PharmML (Pharmacometrics Markup Language)	9/20/2013 12:23 PM
8	VCML	9/4/2013 4:32 PM
9	RDF/XML	9/4/2013 11:49 AM
10	I have been using matlab and c programming	9/4/2013 11:36 AM
11	PSI-MI, XGML	9/4/2013 11:20 AM
12	C++	9/4/2013 11:20 AM
13	example1, example2, example 3	8/2/2013 4:53 PM

Q7 What metadata standards do you use for structuring and annotating your data and models? (Please name all that apply).

Answered: 122 Skipped: 30

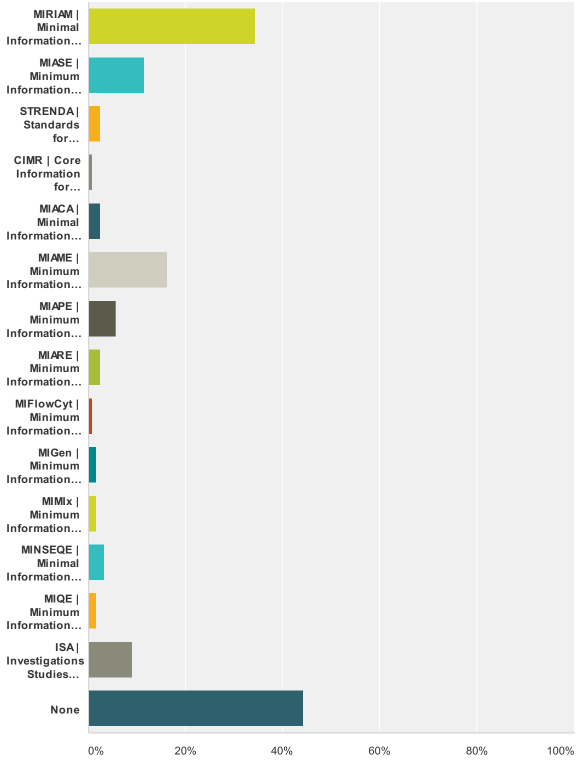

Answer Choices	Responses	
MIRIAM | Minimal Information Required In the Annotation of biochemical Models	34.43%	42
MIASE | Minimum Information About a Simulation Experiment	11.48%	14
STRENDA | Standards for Reporting Enzymology Data	2.46%	3
CIMR | Core Information for Metabolomics Reporting	0.82%	1
MIACA | Minimal Information About a Cellular Assay	2.46%	3
MIAME | Minimum Information About a Microarray Experiment	16.39%	20
MIAPE | Minimum Information About a Proteomics Experiment	5.74%	7
MIARE | Minimum Information About a RNAi Experiment	2.46%	3
MIFlowCyt | Minimum Information for a Flow Cytometry Experiment	0.82%	1
MIGen | Minimum Information About a Genotyping Experiment	1.64%	2
MIMIx | Minimum Information about a Molecular Interaction Experiment	1.64%	2
MINSEQE | Minimal Information about a high throughput SEQuencing Experiment	3.28%	4
MIQE | Minimum Information for Publication of Quantitative Real|Time PCR Experiments	1.64%	2
ISA | Investigations Studies Assays	9.02%	11
None	44.26%	54
Total Respondents: 122		

#	Other (please specify)	Date
1	CellML metadata spec	9/30/2013 10:03 AM
2	MIRIAM intended	9/26/2013 9:50 AM
3	Quatitative approach	9/25/2013 10:38 PM
4	None at this stage, but it will clearly have to change	9/25/2013 3:01 PM
5	MICEE - Minimum information about a Cardiac Electrophysiology Experiment	9/25/2013 2:47 PM
6	Our own Morpheus markup language for multicellular systems biology (to be published soon)	9/25/2013 2:21 PM
7	in so far as the annotations/URIs use identifiers.org	9/19/2013 4:19 PM
8	example 1, example 2	8/2/2013 4:53 PM

ISBE Systems Biology Standards Survey

Q8 What controlled vocabularies and/or ontologies do you use for structuring and annotating your data and models? (Please name all that apply).

Answered: 120 Skipped: 32

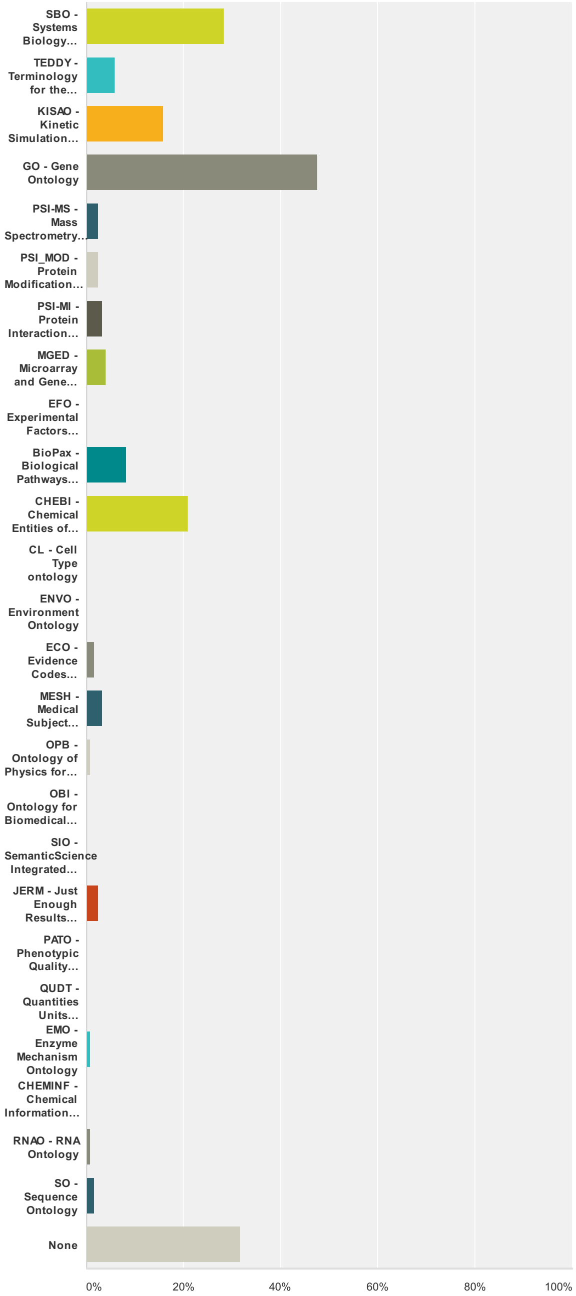

Answer Choices	Responses	
SBO - Systems Biology Ontology	28.33%	34
TEDDY - Terminology for the Description of Dynamics	5.83%	7
KISAO - Kinetic Simulation Algorithm Ontology	15.83%	19
GO - Gene Ontology	47.50%	57
PSI-MS - Mass Spectrometry Ontology	2.50%	3
PSI_MOD - Protein Modification Ontology	2.50%	3
PSI-MI - Protein Interaction Ontology	3.33%	4
MGED - Microarray and Gene Expression Data Ontology	4.17%	5
EFO - Experimental Factors Ontology	0%	0
BioPax - Biological Pathways Ontology	8.33%	10
CHEBI - Chemical Entities of Biological Interest	20.83%	25
CL - Cell Type ontology	0%	0

ENVO - Environment Ontology	**0%**	0
ECO - Evidence Codes Ontology	**1.67%**	2
MESH - Medical Subject Headings	**3.33%**	4
OPB - Ontology of Physics for Biology	**0.83%**	1
OBI - Ontology for Biomedical Investigations	**0%**	0
SIO - SemanticScience Integrated Ontology	**0%**	0
JERM - Just Enough Results Model	**2.50%**	3
PATO - Phenotypic Quality Ontology	**0%**	0
QUDT - Quantities Units Dimensions and Types	**0%**	0
EMO - Enzyme Mechanism Ontology	**0.83%**	1
CHEMINF - Chemical Information Ontology	**0%**	0
RNAO - RNA Ontology	**0.83%**	1
SO - Sequence Ontology	**1.67%**	2
None	**31.67%**	38
**Total Respondents: 120**		

#	Other (please specify)	Date
1	bqbiol; in-house cardiac electrophysiology ontology (under development)	9/30/2013 10:03 AM
2	KEGG	9/26/2013 11:18 AM
3	KEGG	9/25/2013 6:59 PM
4	Currently using whatever ontology supported by semanticSBML 2.0	9/25/2013 3:01 PM
5	Our own Morpheus markup language for multicellular systems biology (to be published soon)	9/25/2013 2:21 PM
6	NeuroLex	9/25/2013 1:22 PM
7	SWO (Software Ontology)	9/20/2013 12:23 PM
8	most of these used through identifiers.org	9/19/2013 4:19 PM
9	EC numbers, KEGG IDs	9/4/2013 11:54 AM
10	example 1, example 2, example 3	8/2/2013 4:53 PM

ISBE Systems Biology Standards Survey

Q9 When searching for data, in which public repositories and data stores do you find useful information? (Please name all that apply).

Answered: 88 Skipped: 64

#	Responses	Date
1	Uniprot	10/7/2013 7:52 AM
2	IntAct, PDB, UniHi, HPRD, BioGRID, BIND, CCSB, DIP, Reactome, ORTHO and others	10/4/2013 11:08 AM
3	ENA, Array Express, GEO, PRIDE, swissprot, KEGG	10/2/2013 6:30 PM
4	Genbank/EMBL RIKEN SRA/ENA/GEO	10/2/2013 12:37 PM
5	brenda	10/2/2013 12:22 PM
6	geo array express mgi string (embl)	10/1/2013 1:43 PM
7	SR, ENA, Array Express	10/1/2013 11:47 AM
8	MINT STRING PhosphoSite	10/1/2013 10:03 AM
9	GEO, TCGA	9/30/2013 9:10 AM
10	NCBI, SRMatlas,	9/26/2013 9:52 PM
11	vitricnexus	9/26/2013 3:53 PM
12	TCGA ICGC BiGG cBIO KEGG GEO HPA	9/26/2013 11:44 AM
13	BioPath.explore; SABIO-RK; KEGG DB; Brenda; Gene Cards	9/26/2013 11:39 AM
14	Too many too mention. NCBI, KEGG, GEO, TCGA...	9/26/2013 11:18 AM
15	UniProt Science Signaling/AAAS KEGG GeneCards PhosphoSitePlus Pathguide	9/26/2013 9:50 AM
16	KeGG, IPA (not public), Uniprot, Human Metabolome database Can it be that you missed the metabolome side of life?	9/26/2013 9:30 AM
17	pubmed, Kegg, expasy, phosphosite plus	9/26/2013 9:27 AM
18	Arrayexpress, GEO, Genebank	9/26/2013 9:23 AM
19	SABIO-RK Brenda Pubmed WoS	9/26/2013 8:43 AM
20	GEO	9/26/2013 8:42 AM
21	www.genedb.org	9/26/2013 7:45 AM
22	GEO	9/26/2013 4:01 AM
23	PubMed, GEO, STRING, BioModels, Phytozome, KEGG	9/25/2013 11:22 PM
24	Biomodels, CellML repository	9/25/2013 11:18 PM
25	GEO, PDB	9/25/2013 9:59 PM
26	Array Express, GEO, GenBank (ncbi.nlm.nih.gov), ENA, EnsEMBL	9/25/2013 7:00 PM
27	Kegg	9/25/2013 6:04 PM
28	KEGG	9/25/2013 5:58 PM
29	Google	9/25/2013 5:57 PM
30	Google	9/25/2013 5:34 PM
31	TAIR NCBI Genbank, GEO BioCyc BioGRID BAR	9/25/2013 5:12 PM
32	KEGG	9/25/2013 4:22 PM
33	SABIO-RK, BRENDA	9/25/2013 3:44 PM
34	mainly via supplementary material or links in publications	9/25/2013 3:20 PM
35	BioModels Database	9/25/2013 3:14 PM
36	Pubmed, GeneDB,TriTrypDB, NCBI	9/25/2013 2:54 PM
37	SabioRK, BiomodelsDB, CellML, ModelDB	9/25/2013 2:51 PM
38	PDB	9/25/2013 2:50 PM
39	pubmed, ncbi, medline	9/25/2013 2:43 PM
40	KEGG, iHOP	9/25/2013 2:21 PM
41	GEO, Array Express, SabioRK, PDB, Genbank, Pedant, SwissProt, UniProt, Brenda, InterPro, Biomodels, JWS online, BIND, MINT, KEGG, Reactome, HUGO, SysMO SEEK	9/25/2013 2:08 PM
42	pubmed	9/25/2013 1:54 PM
43	GEO Cancer Genome Atlas NCBI databases (dbSNP and others)	9/25/2013 1:42 PM
44	SABIO-RK, Brenda	9/25/2013 1:36 PM
45	typically i Start with Google search!	9/25/2013 1:26 PM
46	NIF	9/25/2013 1:22 PM
47	JWS online BioModels	9/25/2013 1:22 PM
48	pubmed	9/25/2013 1:19 PM
49	SabioRK BRENDA IntEnz PubMed	9/25/2013 1:13 PM
50	PDB, KEGG, Genebank	9/25/2013 12:33 PM
51	SABIO-RK	9/20/2013 12:18 PM
52	pathway commons, biomodels.net	9/20/2013 10:03 AM
53	Pubmed, NCBI	9/19/2013 5:48 PM
54	BioModels Datatas SABIO-RK	9/19/2013 11:38 AM
55	biomodels, kegg, sabio rk	9/19/2013 10:32 AM
56	EBI global search	9/19/2013 10:24 AM
57	biomodels database cellml model repository morre (http://sems.uni-rostock.de/projects/morre/)	9/18/2013 11:46 PM
58	Biomodels Bioportal Pubmed KEGG pathways	9/18/2013 9:10 PM
59	KEGG Reactome GEO	9/18/2013 5:29 PM
60	BioModels Database Model Graphs SABIO-RK JWS Online PMR2 Bioportal	9/18/2013 5:29 PM
61	http://sems.uni-rostock.de/projects/morre/	9/18/2013 5:28 PM
62	uniprot, kegg, brenda, SabioRK	9/18/2013 5:28 PM
63	BioNumbers	9/9/2013 11:48 AM
64	sabio-rk, brenda,string, kegg, uniprot	9/4/2013 7:55 PM
65	HMDB, Uniprot, Kegg, BioModels	9/4/2013 6:52 PM

66	MetaCyc, KEGG, NCBI	9/4/2013 3:05 PM
67	KEGG Ecocyc	9/4/2013 2:18 PM
68	matacyc, kegg	9/4/2013 12:02 PM
69	ArrayExpress GEO	9/4/2013 11:59 AM
70	GEO, TCGA, SRA, Encode	9/4/2013 11:59 AM
71	BRENDA, bionumbers,	9/4/2013 11:54 AM
72	Genego Intact PDB KEGG String UniProt PubMED EntrezGene	9/4/2013 11:49 AM
73	GEO, TCGA	9/4/2013 11:39 AM
74	GEO, brenda	9/4/2013 11:39 AM
75	BIOmodelse GEO Pubmed	9/4/2013 11:37 AM
76	Biomodels	9/4/2013 11:36 AM
77	biomodels database, brenda, bionumbers, pubmed, JSW online, Database of Quantitative Cellular Signalling	9/4/2013 11:35 AM
78	GEO HIV-interaction database String-DB	9/4/2013 11:35 AM
79	BioModelsDB, KEGG, GO	9/4/2013 11:29 AM
80	we don't search for data, we produced them ourselves	9/4/2013 11:27 AM
81	BioModels database, KEGGs	9/4/2013 11:27 AM
82	Uniprotkb, Intact, MINT, DIP, GOA, reactome,	9/4/2013 11:20 AM
83	BioModels	9/4/2013 11:20 AM
84	GO, stanford array express	9/4/2013 11:20 AM
85	TAIR, GEO, ERA, NCBI SRA, Ensembl	9/4/2013 11:20 AM
86	SABIO-RK, BRENDA, UNIPROT, ENSEMBL, KEGG, BIOCYC	9/4/2013 11:18 AM
87	BRENDA, KEGG, EcoCyc	9/4/2013 11:18 AM
88	SABIORK, Uniprot	8/2/2013 4:53 PM

ISBE Systems Biology Standards Survey

Q10 What formats, standards and ontologies have you tried to use, but found unsuitable for your purposes? Please list and give a reason why they were unsuitable.

Answered: 36 Skipped: 116

#	Responses	Date
1	GFF format still variable, when produced with one tool, does not validate with another.	10/2/2013 6:32 PM
2	I prefer flat data structures	10/1/2013 10:04 AM
3	We're working to extend SED-ML (see recent COMBINE talks!).	9/30/2013 10:04 AM
4	SBML does not support electrophysiological models properly, libsbml is sometimes hard to install on Linux platforms	9/30/2013 9:11 AM
5	GO,	9/26/2013 9:54 PM
6	SBML. I like to be free to do changes in my models easily without adding all info.	9/26/2013 11:20 AM
7	One standard that is totally missing in the field is that for metagenome and metaproteome analyses. Did youn mention somewhere the Human metabolome data base?	9/26/2013 9:30 AM
8	none	9/26/2013 9:29 AM
9	n.a.	9/26/2013 7:45 AM
10	EBI, it's too complicated than GEO	9/26/2013 4:05 AM
11	I found SBML limited because I found no way to represent a gene as an object that is manipulable in silico (e.g. mutable / knockoutable, expressable, ...)	9/25/2013 7:03 PM
12	The CellML API was frustrating to work with.	9/25/2013 5:58 PM
13	Cellml, math based, no biology in standard	9/25/2013 5:35 PM
14	I would like to modify genome-scale network, but do not know which software could do that	9/25/2013 4:23 PM
15	My data is highly specialized imaging and electrophyiology that doesn't conform to any format. My models are usually composed in VCell or NEURON. VCell permits export of SBML, but the models often contain features that are beyond the SBML specs. Genrally, annotating models is too much trouble and it is not clear how much would really be gained in terms of usage by other platforms.	9/25/2013 3:00 PM
16	GO the terms are hierarchical, resulting in very general and loose terms attached to our group of genes in question, which does not serve finding out a common function of those gens.	9/25/2013 2:57 PM
17	CellML and electrophysiology ontologies need development. There would be very wide applicability.	9/25/2013 2:49 PM
18	some MIBBI standards for experimental data are not "minimum" but maximum information required. In our work we have "reduced" some standards and created templates based on such reduced requirements to make standartisation work more userfriendly	9/25/2013 2:11 PM
19	no such experience	9/25/2013 1:43 PM
20	GO	9/25/2013 1:38 PM
21	slow development times	9/19/2013 10:32 AM
22	CellML Annotation - its just broken	9/18/2013 5:29 PM
23	CellML - problems with the annotation of components;	9/18/2013 5:29 PM
24	SBML GO	9/18/2013 5:29 PM
25	n/a	9/4/2013 5:00 PM
26	SBML level 1, had too limited math possibilities.	9/4/2013 4:33 PM
27	NA	9/4/2013 11:59 AM
28	none as such in my experience as SBML has relatively nice, stable and coherent platform for creating, importing and exporting models.	9/4/2013 11:39 AM
29	None	9/4/2013 11:36 AM
30	NetGenML. I have created a model that is partially supported by Copasi/SBML and partially supported by SPiM (stochastic pi calculus) and almost supported by NFSim/NGML. The main problem is the multi-level structure of the model. As soon as my paper is submitted for publication, I will approach relevant communities to discuss this.	9/4/2013 11:34 AM
31	SBML-Qualitative: I'm working with probabilistic Boolean networks but SBML-Qual doesn't support the integration of probability (if I'm not mistaken). Would be great if I could integrate this information into the model description.	9/4/2013 11:30 AM
32	HL7	9/4/2013 11:23 AM
33	SBML: lack of support for spatial models and dynamical structures.	9/4/2013 11:22 AM
34	None	9/4/2013 11:21 AM
35	SED-ML (parameter estimation not there yet) SBGN (impossible to store rule-based model because of the closed world assumption and the "once a variable always a variable" rule)	9/4/2013 11:19 AM
36	example 1, example 2	8/2/2013 4:54 PM

Q11 Do you find difficulties/problems with reusing or studying existing models?

Answered: 112 Skipped: 40

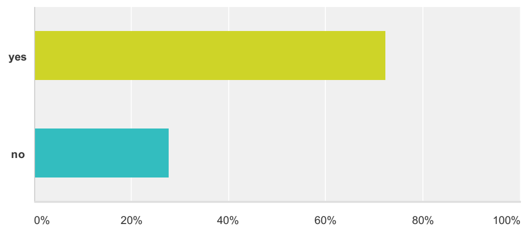

Answer Choices	Responses	
yes	72.32%	81
no	27.68%	31
Total		112

ISBE Systems Biology Standards Survey

Q12 If you answered 'yes' to question 11, what problems do you find?

Answered: 74 Skipped: 78

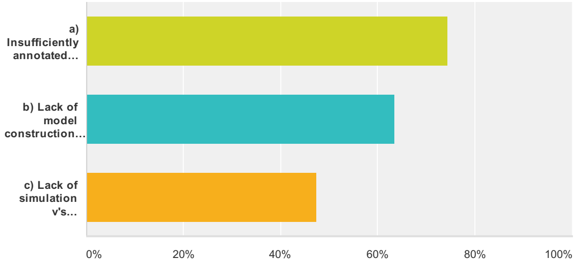

Answer Choices	Responses	
a) Insufficiently annotated models	74.32%	55
b) Lack of model construction and validation data	63.51%	47
c) Lack of simulation v's experimental result comparisons	47.30%	35
Total Respondents: 74		

#	Other (please specify)	Date
1	Lack of standard encoding; errors in encoded version(s)	9/30/2013 10:04 AM
2	Incomplete models - caused by the incorrect or incomplete translation from other model formats to SBML	9/26/2013 11:43 AM
3	Mistakes, bad methods	9/26/2013 11:20 AM
4	Insufficient knowledge/understanding of models on my side...	9/26/2013 9:29 AM
5	Incompatibility between standards versions and tool capabilities (SBML)	9/25/2013 5:14 PM
6	Equation or code does not produce the results shown in the publication	9/25/2013 3:21 PM
7	Models are usually too specialized to be readily reused in another context even if they were properly annotated.	9/25/2013 3:00 PM
8	All of these - in particular lack of model training data.	9/25/2013 2:49 PM
9	mistakes in reactions or parameter spezification	9/25/2013 2:19 PM
10	insufficient description, e.g. parameters missing, initial conditions missing. Note that this problem only exists for models not curated in JWS Online or Biomodels	9/25/2013 1:38 PM
11	not available in standard formats (e.g. SBML)	9/25/2013 1:25 PM
12	lack of meta information (references to other models, relevant versions and update notification, etc)	9/18/2013 11:49 PM
13	errors in text	9/9/2013 11:49 AM
14	technically incorrect model descriptions	9/4/2013 7:57 PM
15	Models do not follow SBML guidelines, manual reformatting required. Running models does not reproduce identical or even comparable results as reported in paper.	9/4/2013 3:07 PM
16	SBML exports from one tool not usable in the SBML-import of another one. (Normally due to loss of data or different styles of encoding those.)	9/4/2013 11:57 AM
17	MISTAKES	9/4/2013 11:40 AM
18	Complex models are inherently difficult, and it is even more difficult to know which parts are realistic.	9/4/2013 11:34 AM

Q13 If you selected 12a, which parts of the model have insufficient annotation?

Answered: 57   Skipped: 95

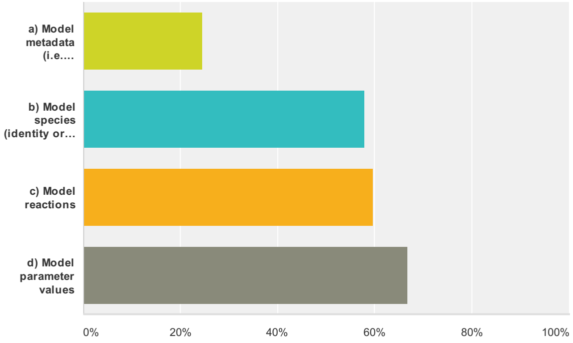

Answer Choices	Responses	
a) Model metadata (i.e. author, purpose, publication)	24.56%	14
b) Model species (identity or names)	57.89%	33
c) Model reactions	59.65%	34
d) Model parameter values	66.67%	38
Total Respondents: 57		

ISBE Systems Biology Standards Survey

Q14 For day-to-day working, where do you store models and data?

Answered: 116 Skipped: 36

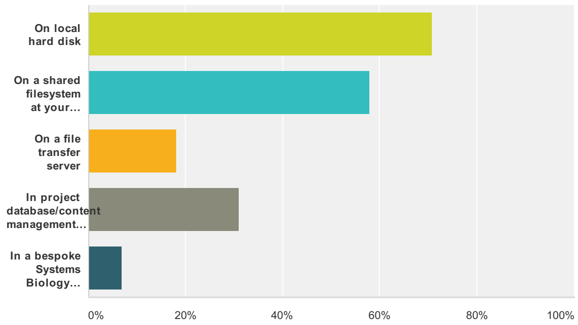

Answer Choices	Responses	
On local hard disk	**70.69%**	82
On a shared filesystem at your institute	**57.76%**	67
On a file transfer server	**18.10%**	21
In project database/content management system	**31.03%**	36
In a bespoke Systems Biology platform	**6.90%**	8
**Total Respondents: 116**		

ISBE Systems Biology Standards Survey

Q15 If you use a project database, content management system, or a bespoke Systems Biology platform, please give the name and a web link.

Answered: 34 Skipped: 118

Answer Choices	Responses	
Systems Biology Platform	97.06%	33
Web Link	91.18%	31

#	Systems Biology Platform	Date
1	Sysmo SEEK	10/7/2013 7:55 AM
2	EUROSPINdb	10/4/2013 11:16 AM
3	Chaste repository	9/30/2013 10:06 AM
4	pyMantis	9/30/2013 9:12 AM
5	SEEK	9/26/2013 9:03 AM
6	SEEK	9/26/2013 7:47 AM
7	SBP	9/26/2013 4:07 AM
8	Mercurial and Git	9/25/2013 11:19 PM
9	We are developing a platform for exploring expression in context of interaction models	9/25/2013 5:16 PM
10	SEEK	9/25/2013 4:16 PM
11	SysMO SEEK	9/25/2013 3:52 PM
12	SysMO-SEEK	9/25/2013 3:49 PM
13	Virtual Cell	9/25/2013 3:00 PM
14	SEEK	9/25/2013 2:59 PM
15	gene explain	9/25/2013 2:46 PM
16	SysMO SEEK	9/25/2013 2:13 PM
17	SEEK	9/25/2013 2:04 PM
18	SEEK	9/25/2013 1:46 PM
19	SEEK, JWS Online	9/25/2013 1:40 PM
20	SEEK VLN	9/25/2013 1:32 PM
21	SEEK	9/25/2013 1:29 PM
22	SEEK	9/25/2013 1:26 PM
23	Open Source Brain	9/25/2013 1:24 PM
24	The SEEK	9/20/2013 12:24 PM
25	SEEK	9/20/2013 12:22 PM
26	Virtual Cell Database	9/20/2013 10:10 AM
27	BioUML	9/19/2013 5:51 PM
28	Biomodels Database	9/19/2013 10:30 AM
29	morre, budhat	9/18/2013 11:51 PM
30	Model Graphs, in-house solution, developed at Rostock University	9/18/2013 5:31 PM
31	Morre	9/18/2013 5:30 PM
32	SysMO SEEK	9/5/2013 1:26 AM
33	Dropbox	9/4/2013 12:22 PM
**#**	**Web Link**	**Date**
1	https://seek.sysmo-db.org	10/7/2013 7:55 AM
2	https://eurospindb.genes2cognition.org/	10/4/2013 11:16 AM
3	https://chaste.cs.ox.ac.uk/trac	9/30/2013 10:06 AM
4	http://rumo.biologie.hu-berlin.de/pyMantis/	9/30/2013 9:12 AM
5	https://seek.sysmo-db.org/	9/26/2013 7:47 AM
6	http://sbp.qfab.org/	9/26/2013 4:07 AM
7	http://mercurial.selenic.com/ http://git-scm.com/	9/25/2013 11:19 PM
8	www.bioinformatics.org/ideas	9/25/2013 5:16 PM
9	http://seek.virtuelle-leber.de/	9/25/2013 4:16 PM
10	https://seek.sysmo-db.org/	9/25/2013 3:52 PM
11	https://seek.sysmo-db.org/	9/25/2013 3:49 PM
12	vcell.org	9/25/2013 3:00 PM
13	seek.sysmo-db.org	9/25/2013 2:59 PM
14	https://seek.sysmo-db.org/	9/25/2013 2:13 PM
15	http://seek.virtuelle-leber.de	9/25/2013 2:04 PM
16	http://seek.virtuelle-leber.de/	9/25/2013 1:46 PM
17	http://seek.sysmo-db.org, http://jjj.mib.ac.uk	9/25/2013 1:40 PM
18	http://seek.virtuelle-leber.de/data_files	9/25/2013 1:32 PM
19	http://seek.virtuelle-leber.de/	9/25/2013 1:29 PM
20	https://seek.sysmo-db.org/	9/25/2013 1:26 PM
21	http://www.opensourcebrain.org	9/25/2013 1:24 PM
22	http://seek4science.org	9/20/2013 12:24 PM
23	seek.virtual-liver.de	9/20/2013 12:22 PM
24	http://vcell.org	9/20/2013 10:10 AM
25	www.biouml.org	9/19/2013 5:51 PM
26	http://www.ebi.ac.uk/biomodels	9/19/2013 10:30 AM
27	http://sems.uni-rostock.de/projects/morre/ - http://budhat.sems.uni-rostock.de/	9/18/2013 11:51 PM
28	http://sems.uni-rostock.de/projects/morre/	9/18/2013 5:31 PM

29	http://sems.uni-rostock.de/projects/morre/	9/18/2013 5:30 PM
30	https://seek.sysmo-db.org/	9/5/2013 1:26 AM
31	I am currently using Microsoft SkyDrive and Office 365, but a web link is not releant because it is currently only accessible for immediate colleagues. I plan to expand access and use other repositories when the appropriate time comes.	9/4/2013 11:37 AM

ISBE Systems Biology Standards Survey

Q16 How do you share models and data with your collaborators before publication?

Answered: 111   Skipped: 41

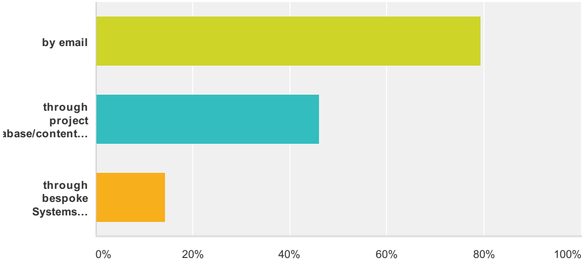

Answer Choices	Responses	
by email	79.28%	88
through project database/content management system	45.95%	51
through bespoke Systems Biology platform	14.41%	16
Total Respondents: 111		

#	Other (please specify)	Date
1	Dropbox	10/4/2013 11:16 AM
2	shared access file system	10/2/2013 6:35 PM
3	FTP	10/1/2013 11:48 AM
4	local repository	9/26/2013 11:47 AM
5	Dropbox	9/26/2013 11:24 AM
6	DropBox	9/26/2013 8:45 AM
7	dropbox.com	9/26/2013 7:47 AM
8	subversion	9/25/2013 7:06 PM
9	SVN; google docs	9/25/2013 5:59 PM
10	file transfer server, and sometime dropbox / owncloud	9/25/2013 2:23 PM
11	dropbox	9/25/2013 1:20 PM
12	Virtual Liver SEEK	9/25/2013 12:23 PM
13	VCell Database currently accessible through Java client only	9/20/2013 10:10 AM
14	dropbox	9/19/2013 10:33 AM
15	SVN	9/19/2013 10:30 AM
16	Google Docs	9/18/2013 5:30 PM
17	Dropbox	9/12/2013 1:48 PM
18	cloud, shared repositories	9/4/2013 11:22 AM

ISBE Systems Biology Standards Survey

Q17 What system(s), if any, do you use to connect experimental data, models and simulation?

Answered: 46 Skipped: 106

#	Responses	Date
1	SEEK	10/7/2013 7:55 AM
2	spreadsheets	10/2/2013 12:24 PM
3	Our functional curation software; Matlab scripts (!)	9/30/2013 10:06 AM
4	R, Matlab	9/30/2013 9:12 AM
5	SBMLSimulator	9/26/2013 11:47 AM
6	R, matlab	9/26/2013 11:24 AM
7	D2D Software	9/26/2013 9:03 AM
8	MAC	9/26/2013 9:03 AM
9	JWS	9/26/2013 7:47 AM
10	Windows	9/26/2013 4:07 AM
11	software i develop, MATLAB or Python code	9/25/2013 11:24 PM
12	transsys / SimGenex	9/25/2013 7:06 PM
13	IDEAs platform mentioned above	9/25/2013 5:16 PM
14	SysMO-SEEK	9/25/2013 3:49 PM
15	AMIGO toolbox; COPASI	9/25/2013 3:15 PM
16	Built in feature of VCell	9/25/2013 3:00 PM
17	SysMO SEEK	9/25/2013 2:59 PM
18	Working on SED-ML improvments for this.	9/25/2013 2:50 PM
19	Matlab	9/25/2013 2:21 PM
20	SysMO SEEK	9/25/2013 2:13 PM
21	JWS Online, SEEK	9/25/2013 1:40 PM
22	Matlab / Copasi	9/25/2013 1:29 PM
23	Vanted (www.vanted.org)	9/25/2013 1:27 PM
24	SEEK	9/25/2013 1:26 PM
25	https://github.com/vellamike/neurotune	9/25/2013 1:24 PM
26	Copasi	9/25/2013 1:16 PM
27	PDB	9/25/2013 12:35 PM
28	The SEEK	9/20/2013 12:24 PM
29	Virtual Cell (vcell.org)	9/20/2013 10:10 AM
30	BioUML	9/19/2013 5:51 PM
31	COPASI	9/19/2013 10:33 AM
32	morre	9/18/2013 11:51 PM
33	Model Graphs	9/18/2013 5:31 PM
34	Matlab	9/12/2013 1:48 PM
35	SEEK	9/5/2013 1:26 AM
36	Copasi	9/4/2013 7:59 PM
37	Per project customised software	9/4/2013 6:57 PM
38	Matlab	9/4/2013 5:01 PM
39	E-cell	9/4/2013 2:19 PM
40	GINsim for model and simulation, Celldesigner for network and detailed annotation of the reactions and publications used	9/4/2013 11:55 AM
41	COPASI and MATLAB independently and in combinations depending on the problem type	9/4/2013 11:45 AM
42	matlab	9/4/2013 11:41 AM
43	-	9/4/2013 11:32 AM
44	word document	9/4/2013 11:29 AM
45	L-studio/vlab	9/4/2013 11:24 AM
46	none	8/2/2013 4:55 PM

Q18 In your experimental work, do you use Standard Operating Procedures?

Answered: 84 Skipped: 68

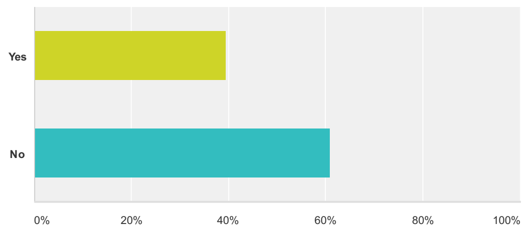

Answer Choices	Responses	
Yes	39.29%	33
No	60.71%	51
Total		84

ISBE Systems Biology Standards Survey

Q19 If you use SOPs, where do you store them and how do you manage them?

Answered: 33 Skipped: 119

#	Responses	Date
1	SEEK Institute Network	10/7/2013 7:55 AM
2	We store them on a data server and manage them just through simple data folders.	10/4/2013 11:16 AM
3	sometimes us them but takes time for them to be formulated and to evolve. store on shared project file system or in project-based system	10/2/2013 6:35 PM
4	computer	10/2/2013 12:24 PM
5	Lab Servers	10/1/2013 11:48 AM
6	file server	10/1/2013 10:04 AM
7	project management system	9/30/2013 9:12 AM
8	Project Database	9/26/2013 9:55 PM
9	I don't remember, do not do experimental work anymore.	9/26/2013 11:24 AM
10	local hard disks	9/26/2013 9:30 AM
11	NA	9/26/2013 4:07 AM
12	SEEK	9/25/2013 6:05 PM
13	SEEK	9/25/2013 6:00 PM
14	Wiki, managed by originator or current users	9/25/2013 5:16 PM
15	lab internal	9/25/2013 4:16 PM
16	SysMO-SEEK	9/25/2013 3:49 PM
17	the cricitical operating procedures are followed blindly with sometimes minor new laboratory adaptations	9/25/2013 2:46 PM
18	SysMO SEEK	9/25/2013 2:13 PM
19	In the project database management system: SEEK	9/25/2013 2:04 PM
20	SEEK Shared filesystem at the Institute	9/25/2013 1:46 PM
21	on shared filesystem of the institute	9/25/2013 1:46 PM
22	SEEK	9/25/2013 1:40 PM
23	Server of research group	9/25/2013 1:32 PM
24	SEEK	9/25/2013 1:26 PM
25	SEEK	9/25/2013 1:16 PM
26	SEEK	9/5/2013 1:26 AM
27	n/a	9/4/2013 5:01 PM
28	Departmental Intranet server	9/4/2013 3:08 PM
29	SOP department	9/4/2013 12:01 PM
30	the operating software (commercial) stores and manages SOPs	9/4/2013 11:55 AM
31	Local HDD	9/4/2013 11:37 AM
32	-	9/4/2013 11:32 AM
33	hard disk	8/2/2013 4:55 PM
